# ID 199 - A Importância do Fomento à Pesquisa Científica, pelo Ministério da Saúde, para Subsidiar a Tomada de Decisão sobre a Incorporação de Produtos de Terapia Avançada no Sistema Único de Saúde

**DOI:** 10.5327/2237-9622.2025.v34s1.199

**Published:** 2025-11-25

**Authors:** Mariella Guimarães Lacerda, Sabrina Moreira Ottani, Jéssica da Silva Rodrigues

**Keywords:** Sistema Único de Saúde, Ministério da Saúde, produtos de terapia avançada, Proadi-SUS, incorporação de tecnologias de saúde

## Abstract

**Introdução:**

O Ministério da Saúde (MS) tem assumido um papel relevante no fomento de pesquisas científicas que visam à realização de ensaios clínicos com produtos de terapia avançada (PTA). Há uma grande expectativa em relação ao uso desses produtos, classificados como medicamentos biológicos, na terapêutica de doenças complexas, raras, sem opções de tratamento, ou em pacientes não responsivos às terapias disponíveis. Atualmente, um dos maiores desafios é avaliar se a incorporação dos PTA ao Sistema Único de Saúde (SUS) é economicamente viável, já que se trata de produtos de alto custo. Nesse contexto, destaca-se a importância do fomento às pesquisas com PTA que, para além da avaliação da segurança, eficácia e efetividade do uso desses produtos, também visam à avaliação dos custos relacionados ao desenvolvimento, à manufatura e ao controle de qualidade dos PTA. Assim, a expectativa é que os resultados dessas pesquisas subsidiem a tomada de decisão sobre a futura incorporação dessas novas tecnologias ao SUS. O objetivo deste trabalho foi realizar um levantamento do total de recursos financeiros investidos pelo MS, por meio do Programa de Apoio ao Desenvolvimento Institucional do Sistema Único de Saúde (Proadi-SUS), em pesquisas científicas que visam à realização de ensaios clínicos com PTA, entre os anos de 2018 e 2024.

**Método:**

Trata-se de um estudo descritivo, baseado na análise de documentos técnicos internos e bases de dados do Departamento de Ciência e Tecnologia, da Secretaria de Ciência, Tecnologia e Inovação e do Complexo Econômico-Industrial da Saúde, do Ministério da Saúde (Decit/Sectics/MS). Para atender ao objetivo, selecionaram-se as pesquisas que cumpriam, concomitantemente, os seguintes critérios: i) ter sido aprovada pelo Decit/Sectics/MS, entre os anos de 2018 e 2024, no âmbito do Proadi-SUS; ii) envolver o desenvolvimento de ensaio clínico com PTA; e iii) visar à futura incorporação da tecnologia ao SUS. O recorte temporal de 2018 a 2024 foi escolhido considerando a publicação, em 2018, pela Agência Nacional de Vigilância Sanitária (Anvisa), do marco regulatório para realização de ensaios clínicos com PTA no Brasil (Resolução da Diretoria Colegiada – RDC – n.º 260, de 21 de dezembro de 2018, revogada pela RDC n.º 506, de 27 de maio de 2021). Por se tratar de produtos de alto custo, de manufatura complexa e que exige mão de obra altamente especializada, optou-se pela seleção de projetos desenvolvidos no âmbito do Proadi-SUS, Programa no qual apenas Entidades de Saúde de Reconhecida Excelência pelo MS podem apresentar propostas.

**Resultados:**

O MS investiu, via Proadi-SUS, entre os anos de 2018 e 2024, um total de R$ 84.490.088,46 em pesquisas com o objetivo de desenvolver ensaios clínicos de fase I com PTA para tratamento de neoplasias hematológicas refratárias/recidivadas, bem como no pós-transplante de medula óssea. A importância do investimento em pesquisas nessa temática vai ao encontro dos resultados de um estudo recente que mostrou que a estimativa de impacto orçamentário no SUS da incorporação de 15 PTA, já disponíveis internacionalmente, variaria entre R$ 16,7 bilhões e R$ 53,2 bilhões (Araújo ., 2024). Esses resultados refletem a relevância do fomento às pesquisas com PTA para embasar discussões acerca de sua incorporação ao SUS.

**Conclusão:**

Os resultados dos estudos mostram que o MS vem investindo recursos consideráveis em pesquisas com PTA. Espera-se que esses resultados apoiem a tomada de decisão baseada em evidências científicas e econômicas sobre a futura incorporação dessas novas tecnologias ao SUS, bem como a seleção de agendas prioritárias e a formulação de novas políticas de saúde.

